# Nuclear Overhauser Enhancement Mediated Chemical Exchange Saturation Transfer Imaging at 7 Tesla in Glioblastoma Patients

**DOI:** 10.1371/journal.pone.0104181

**Published:** 2014-08-11

**Authors:** Daniel Paech, Moritz Zaiss, Jan-Eric Meissner, Johannes Windschuh, Benedikt Wiestler, Peter Bachert, Jan Oliver Neumann, Philipp Kickingereder, Heinz-Peter Schlemmer, Wolfgang Wick, Armin Michael Nagel, Sabine Heiland, Mark Edward Ladd, Martin Bendszus, Alexander Radbruch

**Affiliations:** 1 Department of Neuroradiology, University of Heidelberg Medical Center, Heidelberg, Germany; 2 Neurooncologic Imaging, Department of Radiology, Deutsches Krebsforschungszentrum (DKFZ), Heidelberg, Germany; 3 Department of Medical Physics in Radiology, Deutsches Krebsforschungszentrum (DKFZ), Heidelberg, Germany; 4 Department of Neurooncology, University of Heidelberg Medical Center, Heidelberg, Germany; 5 Department of Neurosurgery, University of Heidelberg Medical Center, Heidelberg, Germany; 6 Department of Radiology, Deutsches Krebsforschungszentrum (DKFZ), Heidelberg, Germany; University of Manchester, United Kingdom

## Abstract

**Background and Purpose:**

Nuclear Overhauser Enhancement (NOE) mediated chemical exchange saturation transfer (CEST) is a novel magnetic resonance imaging (MRI) technique on the basis of saturation transfer between exchanging protons of tissue proteins and bulk water. The purpose of this study was to evaluate and compare the information provided by three dimensional NOE mediated CEST at 7 Tesla (7T) and standard MRI in glioblastoma patients.

**Patients and Methods:**

Twelve patients with newly diagnosed histologically proven glioblastoma were enrolled in this prospective ethics committee–approved study. NOE mediated CEST contrast was acquired with a modified three-dimensional gradient-echo sequence and asymmetry analysis was conducted at 3.3ppm (B1 = 0.7 µT) to calculate the magnetization transfer ratio asymmetry (MTR_asym_). Contrast enhanced T1 (CE-T1) and T2-weighted images were acquired at 3T and used for data co-registration and comparison.

**Results:**

Mean NOE mediated CEST signal based on MTR_asym_ values over all patients was significantly increased (p<0.001) in CE-T1 tumor (−1.99±1.22%), tumor necrosis (−1.36±1.30%) and peritumoral CEST hyperintensities (PTCH) within T2 edema margins (−3.56±1.24%) compared to contralateral normal appearing white matter (−8.38±1.19%). In CE-T1 tumor (p = 0.015) and tumor necrosis (p<0.001) mean MTR_asym_ values were significantly higher than in PTCH. Extent of the surrounding tumor hyperintensity was smaller in eight out of 12 patients on CEST than on T2-weighted images, while four displayed at equal size. In all patients, isolated high intensity regions (0.40±2.21%) displayed on CEST within the CE-T1 tumor that were not discernible on CE-T1 or T2-weighted images.

**Conclusion:**

NOE mediated CEST Imaging at 7T provides additional information on the structure of peritumoral hyperintensities in glioblastoma and displays isolated high intensity regions within the CE-T1 tumor that cannot be acquired on CE-T1 or T2-weighted images. Further research is needed to determine the origin of NOE mediated CEST and possible clinical applications such as therapy assessment or biopsy planning.

## Introduction

Magnetic resonance imaging (MRI) has become the gold standard for the assessment of intracerebral lesions and is thus the primary tool for diagnosis and follow up examination of glioblastoma [1]. Within clinical routine, diagnosis of glioblastoma is usually based on T1-weighted gadolinium contrast enhanced MRI (CE-T1) and T2-weighted images. The limitation of this approach is that CE-T1 images exclusively visualize the disruption of the blood brain barrier and hence lack identification of non-enhancing tumor portions [2], [3]. Furthermore, T2-weighted images cannot distinguish between infiltrative tumor growth and other possible causes of non-specific T2-signal increases [4]. Therefore, alternative sequences for the determination of the most malignant tumor parts and the tumor extent are highly desirable.

Chemical Exchange Saturation Transfer (CEST) imaging is a non-invasive MRI technique sensitive to endogenous mobile proteins and peptides respectively and their tissue specific concentration [5], [6]. Multiple metabolites (e.g. glutamate, creatine, myo-inositol, proteins) possess exchangeable protons and thus become endogenous agents with distinct chemical shifts making CEST a technology with the potential for frequency selective molecular imaging [5]. Signal contrast related to mobile proteins results from saturation of their exchanging protons by selective radiofrequency irradiation. Protons in the saturated state transfer to free bulk water yielding a reduction of local z-magnetization of water protons. This leads to a successive signal reduction in the water pool allowing indirect MR imaging of mobile proteins.

Biomedical applications have for example been demonstrated for the detection and grading of tumors [7], [8], [9], [10], the differentiation between tumor progress and radiation necrosis [11] and acute stroke imaging [12].

At low saturation power (e.g. 0.6–0.8 µT) CEST studies revealed that saturation transfer at −2 to −5 ppm is predominantly mediated by Nuclear Overhauser Enhancement (NOE) effects [13], [14], [15]. NOE mediated CEST effects are attributed to aliphatic and olefinic protons in mobile proteins [14]. Initial examinations of human brain tumors at 7 Tesla (7T) showed for one patient with astrocytoma WHO Grade III [14] and one patient with glioblastoma [15] that NOE mediated CEST effects significantly drop in tumor tissue.

In the current study, we investigated if NOE-weighted CEST-MRI with high 3D spatial resolution at 7T and precise sequence co-registration provides additional information about glioblastoma imaging, specifically the visualization of surrounding tumor hyperintensities and isolated CEST high intensity regions (HIR) that do not display on CE-T1 or T2-weighted images.

## Patients and Methods

### Patients

Twelve patients (3 female, 9 male; age: 62.58±12.67 years) with newly diagnosed and subsequently histopathologically confirmed glioblastoma were included in this prospective study. The study was approved by the Medical Ethics Committee (Faculty of Clinical Medicine, University of Heidelberg, Germany) and written informed consent was received from all participants before enrollment.

### Conventional MRI at 3T

CE-T1 weighted (TE = 4.04 ms, TR = 1710 ms, FoV 256×256, resolution 512×512, slice thickness 1 mm) and T2-weighted (TE = 89 ms, TR = 5140 ms, FoV 172×229, resolution 384×230, slice thickness 4 mm) images were acquired on a 3T whole body MR imaging system (Magnetom Verio/Trio TIM; Siemens Healthcare, Erlangen, Germany).

### CEST-MRI at 7T

The CEST sequence was performed on a 7T whole body MRI scanner (Magnetom 7T; Siemens Healthcare, Erlangen, Germany) with a time delay of 1–5 days in relation to 3T MRI. CEST imaging was performed with a centric-reordered three-dimensional gradient echo sequence [16] with the following parameters: Base resolution 128, FoV phase = 78.125%, 26 slices, resolution = [1.8 mm×1.8 mm]×2 mm, TR = 12 ms, TE = 2.88 ms, BW = 320 HZ/px, FA = 10°, GRAPPA acceleration factor 3. Before each segment of the 3D stack a saturation pulse train was applied. The pulse train consisted of 5 gaussian pulses with a duration of 100 ms per pulse and a pulse-train-average amplitude of B_1_ = 0.7 µT. Due to SAR limitations, the interpulse delay was 100 ms leading to an effective saturation time of 900 ms. For each pixel the reduced water magnetization M was normalized by the unsaturated magnetization M_0_ yielding Z = M/M_0_. Z plotted as a function of the irradiation frequency offset Δω relative to the water resonance formed the Z-spectrum. Thirteen equidistant frequency offsets between −4 and +4 ppm and the additional M_0_ image were acquired, resulting in an acquisition time of 9 min 30 s. Due to B0 inhomogeneities, irradiation frequency offsets are distorted, but can be corrected by post-processing [16], [17], [18] using a B0 map. Similar to Stancanello et al. [18] we obtained a B0 map by determining the minima of the Z-spectra for each pixel employing a cubic spline interpolation. Consequently, every Z-spectrum was interpolated and shifted to assure that the minimum of the Z-spectrum, and thus the water resonance, is at 0 ppm. CEST signal intensity was defined by the magnetization transfer ratio asymmetry (MTR_asym_) at 3.3 ppm and B_1_ = 0.7 µT, which was calculated pixel-wise by MTR_asym_(3.3 ppm, B_1_ = 0.7 µT) = Z(Δω = −3.3 ppm, B_1_ = 0.7 µT) - Z(Δω = +3.3 ppm, B_1_ = 0.7 µT). The resulting CEST contrast defined by MTR_asym_ thus shows hyperintensities where NOE mediated CEST effects are decreased and vice versa for hypointensities. The CEST contrast was windowed between MTR_asym_ from −10% to 5%. Images of 3T sequences (CE-T1 and T2) were co-registered on CEST MTR_asym_ using a SIEMENS Syngo Fusion Station.

### Qualitative Analysis

Analyses of 3D-coregistered CE-T1, T2-weighted and CEST images were performed by two neuroradiologists (AR and PK). Discrepancies were resolved by consensus reading. CE-T1 tumor and tumor necrosis were identified on CE-T1 and peritumoral edema on T2-weighted images. Size and structure of the edema on T2-weighted images were visually compared to corresponding peritumoral hyperintensities on co-registered CEST images in three dimensions. The appearance of isolated CEST HIR on MTR_asym_ within the area of CE-T1 tumor and within the area of peritumoral edema according to its extent on T2-weighted images were evaluated. Illustration of CEST HIR was performed in the same window (−10% to +5%) but with different color gradients for improved visualization. Finally, the appearance of tumor satellite lesions (defined as contrast enhanced lesions on CE-T1, diameter <1 cm, without connection to the main tumor) was investigated on CE-T1, T2-weighted and on corresponding CEST images.

### Spectral analysis

Six regions of interest (ROI) were selected for each patient on co-registered data in a representative slice for quantitative MTR_asym_ signal analysis. The selection of the ROIs was performed based on best visibility of the several tissues on the following sequences: 1) CE-T1 tumor and 2) tumor necrosis were selected on CE-T1 images. 3) Isolated CEST HIR within the CE-T1 tumor and 4) peritumoral CEST hyperintensites (PTCH) within T2 edema margins were directly selected on MTR_asym_. 5) Cerebrospinal fluid (CSF) and 6) contralateral normal appearing white matter (CLNAWM) were selected on T2-weighted images. For each ROI the average MTR_asym_ was determined. Furthermore, the contribution of up and downfield effects on MTR_asym_ within Z-spectra were visually compared for each ROI.

### Statistical Analysis

The data from ROI analysis was used for statistical evaluation. A repeated measures analysis of variance (rm ANOVA) for all regions and patients and post hoc Holm-Sidac pairwise multiple comparisons were performed with SigmaPlot version 12.5 (Systat Software, Inc., San Jose California USA). The level of significance was set at P<0.05.

## Results

CEST effects given by MTR_asym_ were observed in a minimum-to-maximum range from −25% to +12% resulting from the asymmetry analysis based on the measured Z-spectra. 98.51% of all intracranial values were in the range from −12% to +5%. All tumors could be identified on MTR_asym_ as hyperintense lesions since NOE mediated CEST effects decreased in all glioblastoma tumors. Highest MTR_asym_ intensity values appear in CSF and in isolated CEST HIR of the CE-T1 tumor, both showing MTR_Asym_ values of approximately 0. For CSF this is because no saturation transfer is apparent neither at +3.3 ppm nor at −3.3 ppm. Within the isolated CEST HIR of the CE-T1 tumor, MTR_asym_ = 0 reflects that NOE signals (−3.3 ppm) are of equal size as saturation transfer effects at the opposite side of the Z-spectrum (+3.3 ppm).

### Qualtitative analysis

**Figure 1:**
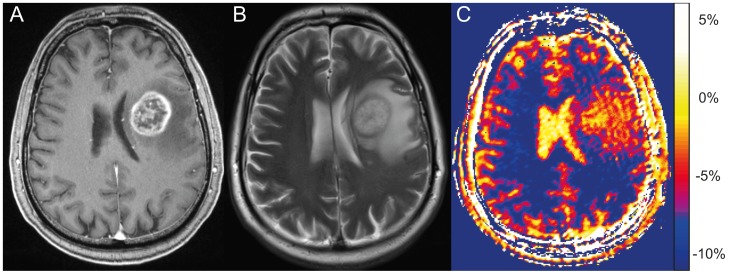
Peritumoral hyperintensity on NOE mediated CEST compared to standard MRI. Left frontal glioblastoma in a 59 year old man at 3 Tesla, CE-T1 (A) and T2-weighted images (B). On the selected slice the CEST contrast at 7 Tesla, based on MTR_asym_ (C), displays peritumoral hyperintensities at equal extent compared to the edema on T2-weighted images. In contrast to T2-weighted images, the CEST peritumoral hyperintensity displays an irregular border and subareas of different signal intensity.

In eight out of 12 patients, the peritumoral hyperintensity on CEST was smaller than on T2-weighted images. In four patients, CEST displayed congruent areas. In two of the eight patients with smaller peritumoral hyperintensity, the CEST hyperintensity moderately exceeded the T2 edema in one direction. In comparison to the edema on T2-weighted sequences, peritumoral hyperintensities on CEST displayed an irregular border and subareas of different signal intensity. Furthermore, stria like structures could be identified on CEST images within peritumoral hyperintensities (Fig. 1).

Tumor necrosis on CEST appeared predominantly hyperintense compared to average signal in peritumoral hyperintensities. Within the corresponding area of CE-T1 tumor, CEST images revealed heterogeneous signal intensities. The MTR_asym_ signal intensity in the CE-T1 tumor varied from isointense to peritumoral hyperintensities to isolated CEST HIR.

Isolated CEST HIR on MTR_asym_ within the CE-T1 tumor could be observed in all 12 patients. In eight patients isolated CEST HIR could be additionally identified within the edema according to its extent on T2-weighted images.

**Figure 2:**
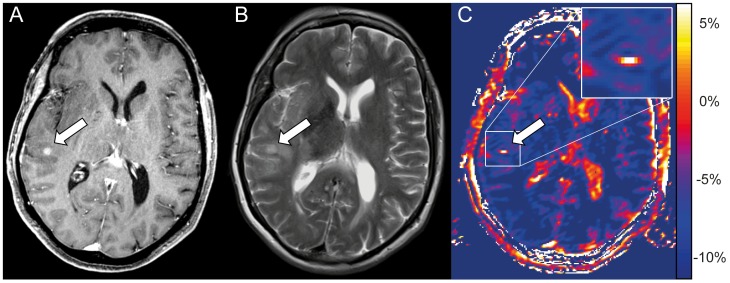
Tumor satellite lesion displays hyperintense on NOE mediated CEST. Tumor satellite of a glioblastoma subcortical temporal right in a 67 year old woman. The satellite presents a clear enhancement on CE-T1 (arrow in A) and barely displays on the T2-weighted image (arrow in B). In contrast, the satellite displays clearly hyperintense on CEST based on MTR_asym_ (C) and matches with the area of contrast enhancement on the CE-T1 image (A). Furthermore also CSF in lateral ventricles and cerebral sulci displays hyperintense on MTR_asym_.

A total of eight tumor satellites were identified in the patient collective on CE-T1. Four of these eight satellites were clearly hyperintense both on T2-weighted images and on CEST, while three of the eight CE-T1 satellites barely displayed on T2-weighted images and were also clearly visible on CEST images (Fig. 2). One tumor satellite was not hyperintese on CEST and barely hyperintense on T2-weighted images.

Table 1 summarizes the observations obtained from qualitative data analyses.

**Table 1:** Qualitative analyses of NOE-mediated CEST contrast on 3D co-registered data.

Patient No.	Size of peritumoral hyperintensity:	Appearance of isolated high intensity regions (HIR) on MTR_asym_ in the area of:		Satellite lesions:
	CEST versus T2-w.	CE–T1 tumor	T2 peritumoral edema	CEST hyperintense/Total on CE-T1
#1	smaller	Y	Y	∅
#2	equal	Y	N	1/1
#3	smaller	Y	N	∅
#4	equal	Y	Y	2/2
#5	smaller	Y	Y	1/1
#6	smaller	Y	Y	∅
#7	smaller	Y	N	∅
#8	smaller	Y	Y	∅
#9	smaller*	Y	N	∅
#10	equal	Y	Y	∅
#11	smaller*	Y	Y	2/3
#12	equal	Y	Y	1/1

Peritumoral hyperintensity: Comparison of the extent of the peritumoral hyperintensity on CEST and T2-weighted images (smaller* = total extent smaller on CEST contrast but exceeding the margins of the T2 edema in one direction). **Appearance of isolated high intensity regions (HIR) on MTR_asym_**: Evaluation if isolated CEST HIR on MTR_asym_ displayed in the area of CE-T1 tumor or T2 peritumoral edema (Y = Yes, N = No). **Satellite lesions**: Fraction of contrast enhanced satellite lesions identified on CE-T1 images that were also clearly hyperintense on CEST (∅ = no satellite lesion detected).

### Spectral analysis

**Figure 3:**
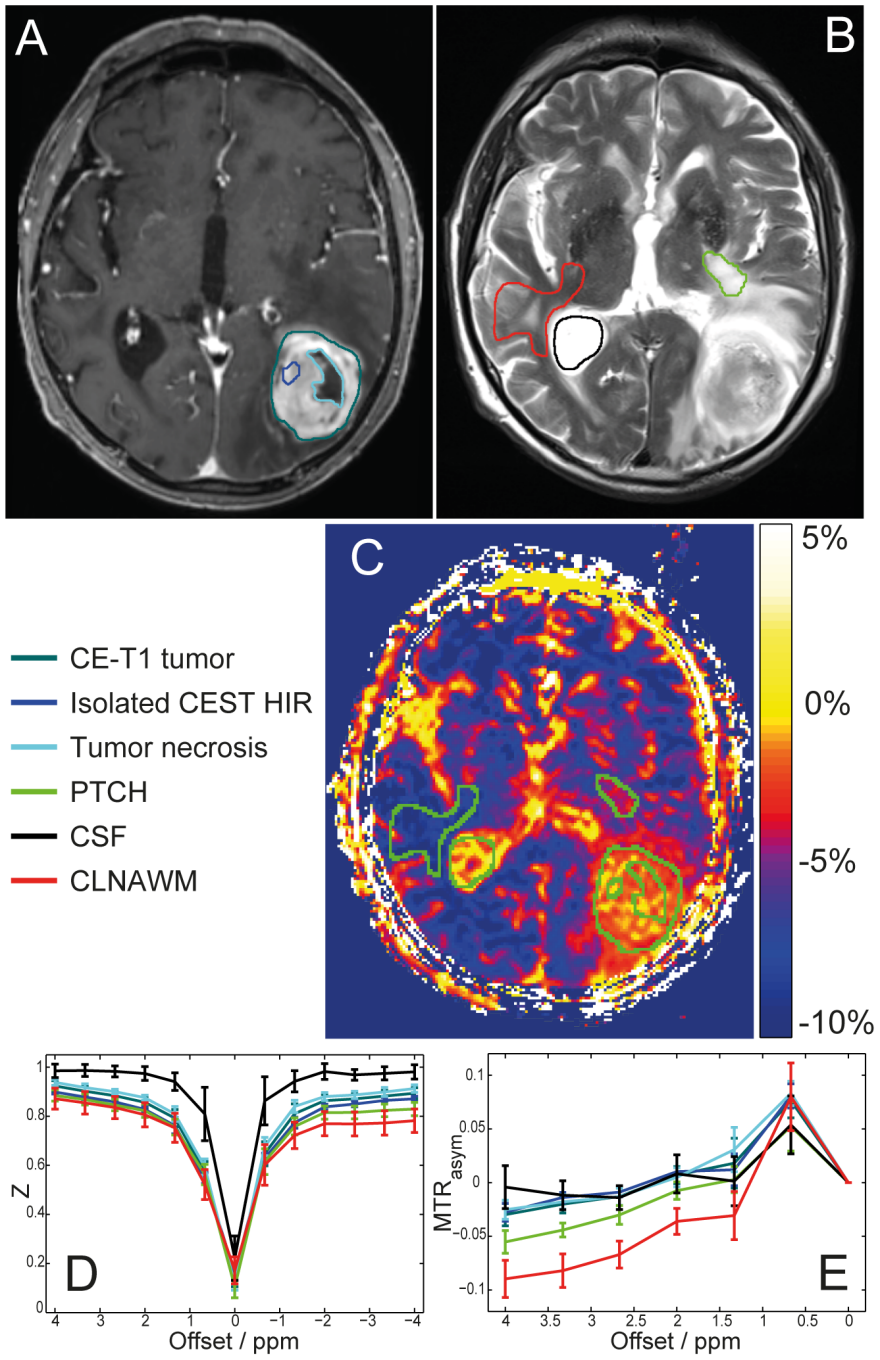
Regions of interest (ROI) selection for spectral analysis and MTR_asym_ quantification. Left occipital glioblastoma of a 79 year old patient, CE-T1 (A) and T2-weighted images (B) with color coded ROIs: CE-T1 tumor, isolated CEST HIR within CE-T1 margins, tumor necrosis, PTCH within T2 edema margins, CSF and CLNAWM. CEST contrast based on MTR_asym_ (C): Same ROIs illustrated in green for improved visualization. Z-spectrum (D) and asymmetry analysis (E) shown. Analyses of Z-spectra reveals that a decrease of NOE upfield effects at −3.3 ppm causes the hyperintense MTR_asym_ contrast in the tumor regions, while no clear APT peak around +3.3 ppm could be identified in any of the analyzed tissues. Even though MTR_asym_ shows high intensities both in CSF and isolated CEST HIR within CE-T1 tumor, Z-spectrum analysis reveals that the underlying asymmetry has a different origin: no saturation transfer is apparent in CSF at ±3.3 ppm (D black line) while in tumor regions (D dark green, dark blue and light blue lines) MTR_asym_ = 0 reflects that NOE signals (−3.3 ppm) and saturation transfer effects at the opposite side of the Z-spectrum (+3.3 ppm) are of equal size. Furthermore the width of the Z-spectrum of CSF is decreased due to the longer T2 relaxation time.

Mean MTR_asym_ in CE-T1 tumor was −1.99±1.22% and −1.36±1.30% in tumor necrosis. For isolated CEST HIR on MTR_asym_ within CE-T1 tumor the mean signal strength was 0.40±2.21% and −3.56±1.24% in PTCH within T2 edema margins. In CSF average MTR_asym_ value was 0.76±1.29% and −8.38±1.19% in CLNAWM (Fig. 3). In the tumor and peritumoral tissue of all patients (CE-T1 tumor, tumor necrosis, PTCH within T2 edema margins and isolated CEST HIR within CE-T1 tumor), a clear decrease of the Z-values around -3.3 ppm was observed compared to CLNAWM. At +3.3 ppm, no clear change in saturation transfer effect could be identified in any tissue and in any patient. A representative Z-spectrum analysis is given in Fig. 3D.

### Statistical Analysis

**Figure 4:**
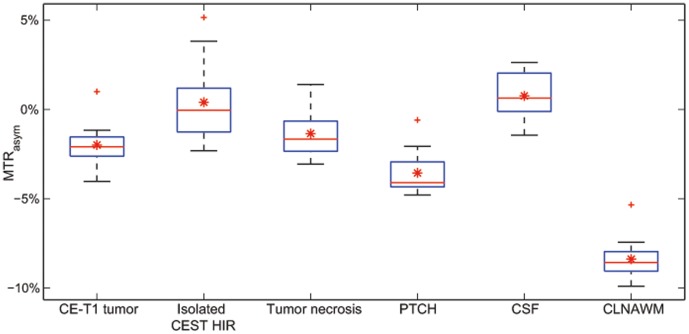
Boxplots of MTR_Asym_ quantification from regions of interest (ROI) analysis over all glioblastoma patients. Boxplots of mean MTR_Asym_ values on CEST contrast over all patients (N = 12). Overall mean MTR_asym_ (red stars) and outliers (red crosses) are additionally illustrated. MTR_asym_ values in all tumor areas (CE-T1 tumor, isolated CEST HIR in CE-T1 tumor, tumor necrosis) and CSF are significantly higher than in CLNAWM (p<0.001). Average signal intensity in PTCH within T2 edema margins is significantly higher (p<0.001) than in CLNAWM and significantly lower (p = 0.015) than in CE-T1 tumor and tumor necrosis (p<0.001). The whiskers of the boxplot for isolated CEST HIR indicate a high variance within this group, which is due to smaller ROI size and the fact that the isolated CEST HIR were visually selected relative to surrounding signal intensity in CE-T1 tumor.

The analysis of variance (ANOVA) with repeated measures was p<0.001 for statistically significant differences among the six groups by ROI analysis. Post hoc Holm-Sidac pairwise multiple comparisons showed that average MTR_asym_ of CE-T1 tumor, isolated CEST HIR within the CE-T1 tumor, tumor necrosis, PTCH within T2 edema margins and CSF were all significantly higher than MTR_asym_ of CLNAWM (p<0.001). Mean MTR_asym_ in PTCH within T2 edema margins was significantly increased (p<0.001) compared to CLNAWM and significantly lower than in CE-T1 tumor (p = 0.015) and tumor necrosis (p<0.001). Average MTR_asym_ in isolated CEST HIR within CE-T1 tumor was significantly higher than in the whole CE-T1 tumor (p<0.001) and PTCH within T2 edema margins (p<0.001). In tumor necrosis, MTR_asym_ was significantly lower than in isolated CEST HIR within CE-T1 tumor (p = 0.007) and CSF (p = 0.001) but not significantly different compared to CE-T1 tumor (p = 0.42) (Fig. 4).

## Discussion

We demonstrated that a contrast in glioblastoma can be obtained by NOE mediated CEST imaging at 7T in terms of structure and extent of peritumoral hyperintensities and isolated CEST HIR that cannot be acquired with conventional CE-T1 and T2-weighted images.

As a principle finding of this study, we proofed, that the NOE-effects in glioblastoma CE-T1 tumors, as well as in the tumor necrosis and the surrounding PTCH within T2 edema margins are decreased in comparison to CLNAWM. This is in agreement with previously published studies [14], [15]. However, the cause of the NOE drop within tumor tissue is still under discussion. Due to the exchange relayed mechanism of NOE, changes in pH may result in altered NOE contrast [22]. Nevertheless, intracellular changes in pH are supposed to be small in gliomas and only a subtle pH increase (up to approximately 0.1 pH) was reported [23], [24]. Since the increase of NOE was shown to be smaller than 0.7% per pH [14], [25] we assume that pH changes are not the dominant origin of the observed effect.

A lowered protein concentration might explain the NOE drop since water content in glioblastoma is supposed to be higher and extravasated serum proteins are reported to be lower than in healthy tissue [26], [27]. Accordingly, the significantly decreased NOE in the CE-T1 tumor compared to the PTCH within T2 edema margins could be explained as protein concentration within the enhancement is supposed to be lower [27]. NOE results from dipolar interactions between protons that highly depend on the molecular structure. Therefore, an additional explanation would be a higher mobility of proteins in tumor tissue which would lower the dipolar cross-relaxation rates. Recently, NOE effects also turned out to be correlated with protein structure [15]. Thus, a further mechanism could be that the protein structure itself is less compact due to increased misfolding of the rapidly produced proteins within the area of highest proliferation. Based on the given data, it is not possible to identify the contribution of each possible cause.

Since NOE mediated CEST imaging provides additional information compared to standard MR sequences, there are numerous possible clinical applications that need to be evaluated. For biopsy guidance, the commonly used CE-T1 images lack specificity, because they only visualize the extravasation of contrast agent due to a disrupted blood brain barrier. Subareas of different signal intensity within glioblastoma on NOE mediated CEST may therefore contribute to identify tumor parts of different malignity by adding information about protein concentration or protein folding.

Since we detected seven out of eight tumor satellites on MTR_asym_ images as hyperintense, an additional use of NOE mediated CEST as endogenous contrast might increase sensitivity for the identification of tumor satellites. However, as CSF in brain sulci and ventricles as well as blood vessels also display hyperintense on MTR_asym_, specificity of NOE mediated CEST is limited and requires comparison to anatomic sequences.

Another major problem in glioblastoma imaging within daily clinical decision making is that it is not possible to differentiate a T2-signal increase caused by tumor infiltration from a non-specific cause of T2-signal increase (e.g. edema, radiation effects, decreased corticosteroid dosing, seizures, postoperative changes) [2], [28]. In this context, CEST images showed peritumoral hyperintensities which tended to be smaller than T2 edema extent and revealed substructures that were not discernible on T2-weighted images. A potential explanation for this finding might be that NOE mediated CEST displays the tumor infiltration more accurately than non-specific T2-weighted images. However, comparing CEST at 7T with T2-weighted images at 3T might be inappropriate, especially concerning heterogeneities within the tumor and its peritumoral edema. Within future examinations, comparisons should be performed with T2-weighted images also acquired at 7T. Furthermore, all assumptions based on variations in signal intensity are hypothetical and should be investigated in animal studies with precise histopathological correlation.

Other CEST studies on high grade glioma patients were performed by Wen et al. [7] and Zhou et. al [10] using APT mediated CEST at 3T based on asymmetry approaches. They reported increased APT effects within the tumor and described peritumoral hyperintensities that also tended to be smaller than on T2 weighted images. In contrast to our NOE weighted approach yielding negative MTR_asym_ values, their APT weighted CEST yielded positive MTR_asym_ values in all tissues. Advantages of the 7T field strength in our study are higher spectral resolution and higher SNR or shorter measurement times, respectively. Consequently APT mediated CEST image contrast between the tumor and non-tumorous parenchyma is only ∼1.5–2% on MTR_asym_ [7] whereas we measured differences of ∼6–8% between CE-T1 tumor and CLNAWM on MTR_asym_. Interestingly, by comparing only changes in asymmetry values, we see the same trend towards higher MTR_asym_ values within the tumor as APT-weighted studies.

To evaluate therapeutic response effects of chemotherapy with temozolomide in mice with human glioblastoma, Sagiyama et al. recently performed asymmetry based APT imaging on a 7T small animal MR system [29]. They were able to show that APT signal in treatment resistant tumors increased significantly within one week compared to tumors that responded to treatment. Furthermore, they found a high correlation between the histopathologically determined KI67 labeling index and APT signal intensity in the tumors. These results indicate that CEST imaging might contribute to differentiate tumor progression from treatment effects in glioma.

Generally, when performing CEST asymmetry approaches, it has to be verified that the implicit assumption of competitive CEST effects is valid. At +3.5 ppm amide proton transfer (APT) of proteins was reported, while exchange relayed NOE occurs in the range from −2 to −5 ppm [6], [19]. In the current study we chose 3.3 ppm to reduce APT effects in the asymmetry analysis. Furthermore, we used a saturation field amplitude of 0.7 µT where NOE effects are much stronger than APT effects that peak at 2.1 µT [13], [15]. These theoretical considerations were confirmed in our spectral analysis since we could identify a clear decrease of effects at −3.3 ppm in all tumor parts compared to CLNAWM, while only small background effects at +3.3 ppm most probably due to exchanging amide protons could be detected. A confounding factor in asymmetry analysis is the semi-solid magnetization transfer. However, for the irradiation scheme used in this study contributing effects should be small (<1–2% in MTR_asym_) since magnetization transfer only becomes dominant for higher irradiation powers [19], [20], [21].

Finally, the clinical benefit of the newly introduced NOE mediated CEST contrast still needs to be proven within larger patient collectives, including follow up examinations and bioptical correlations. Ultimately, beyond MTR_asym_ approaches, sophisticated fitting models performed on Z-spectra with additional frequency offsets may help to separate competitive CEST effects yielding a metabolite specific CEST contrast.
